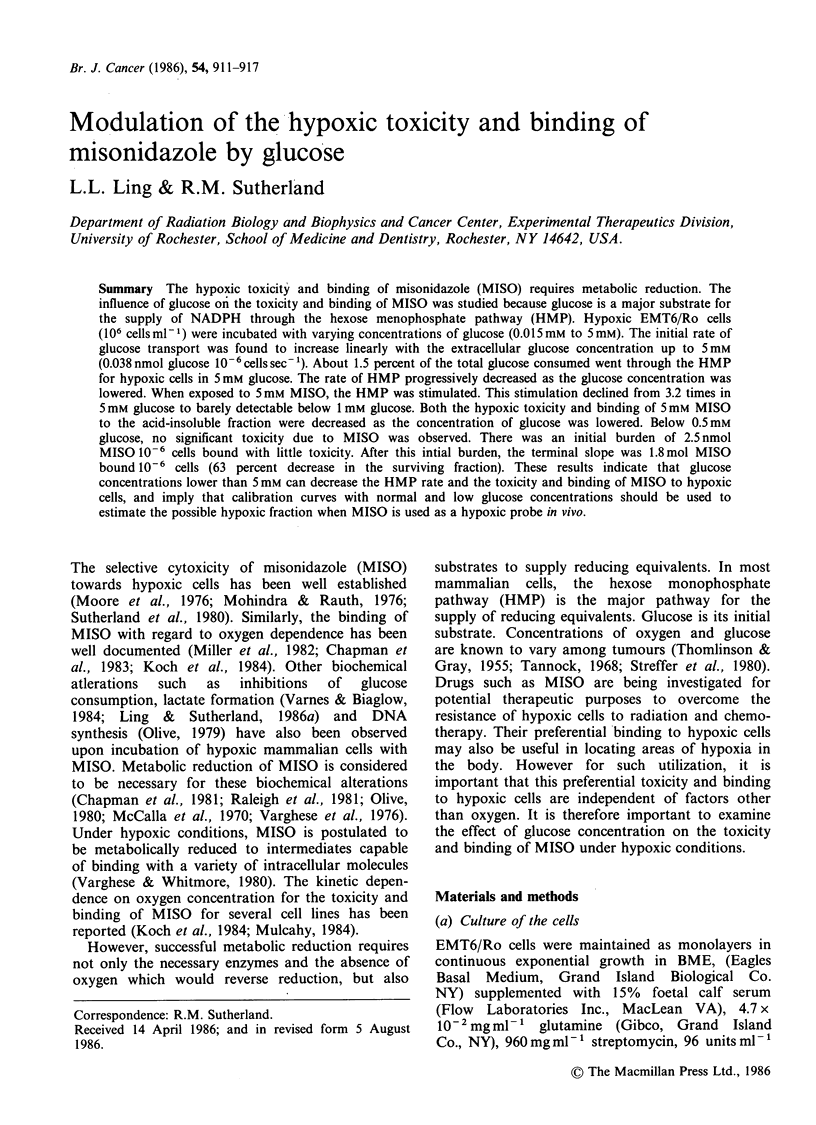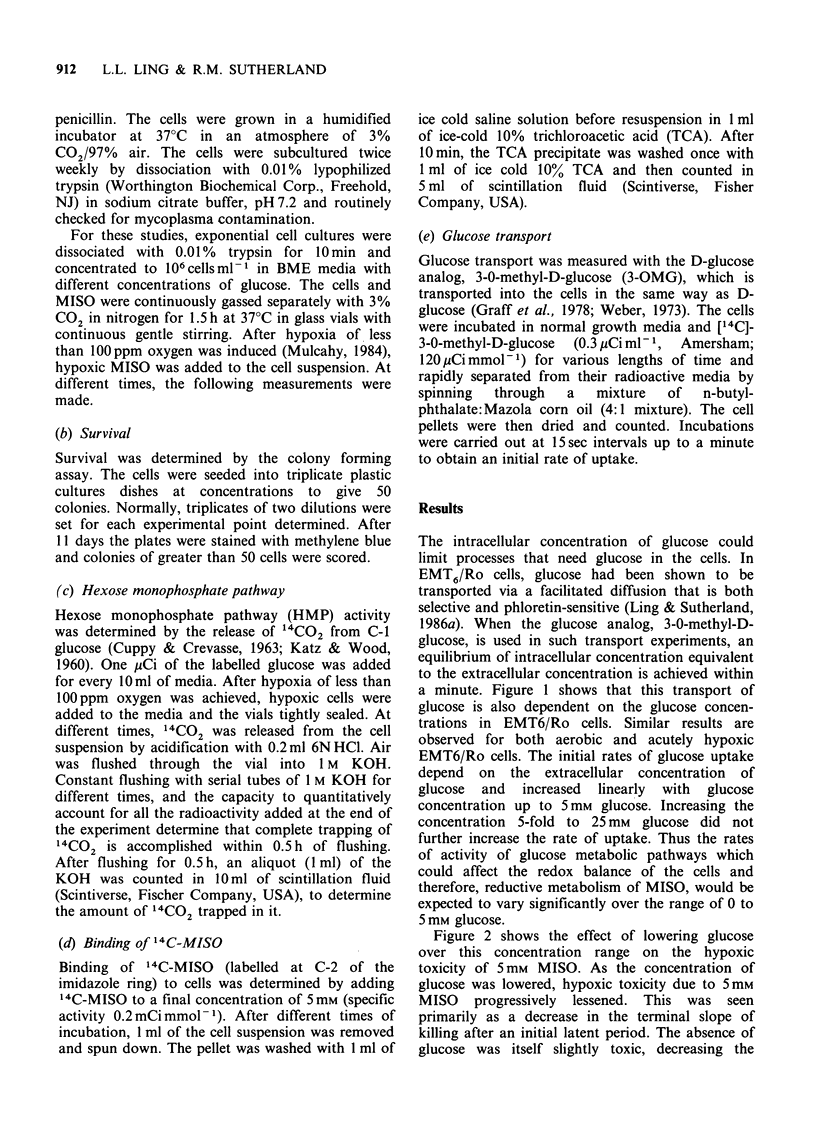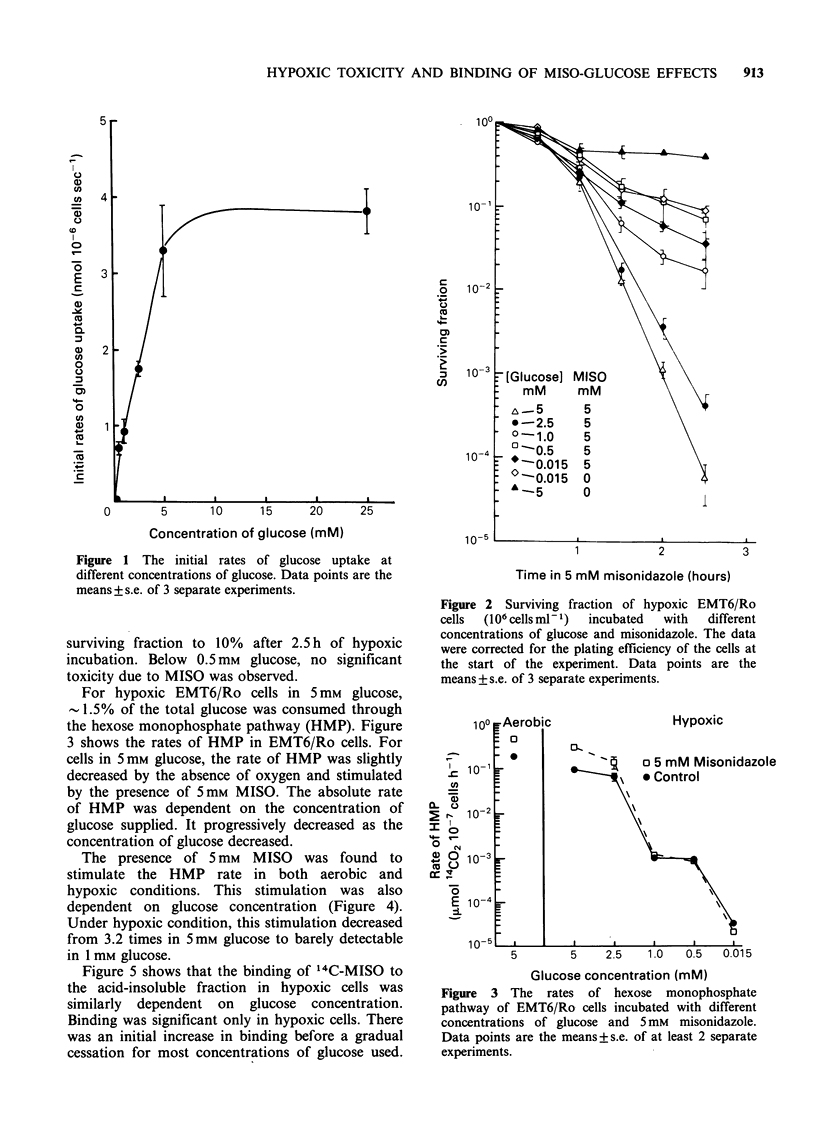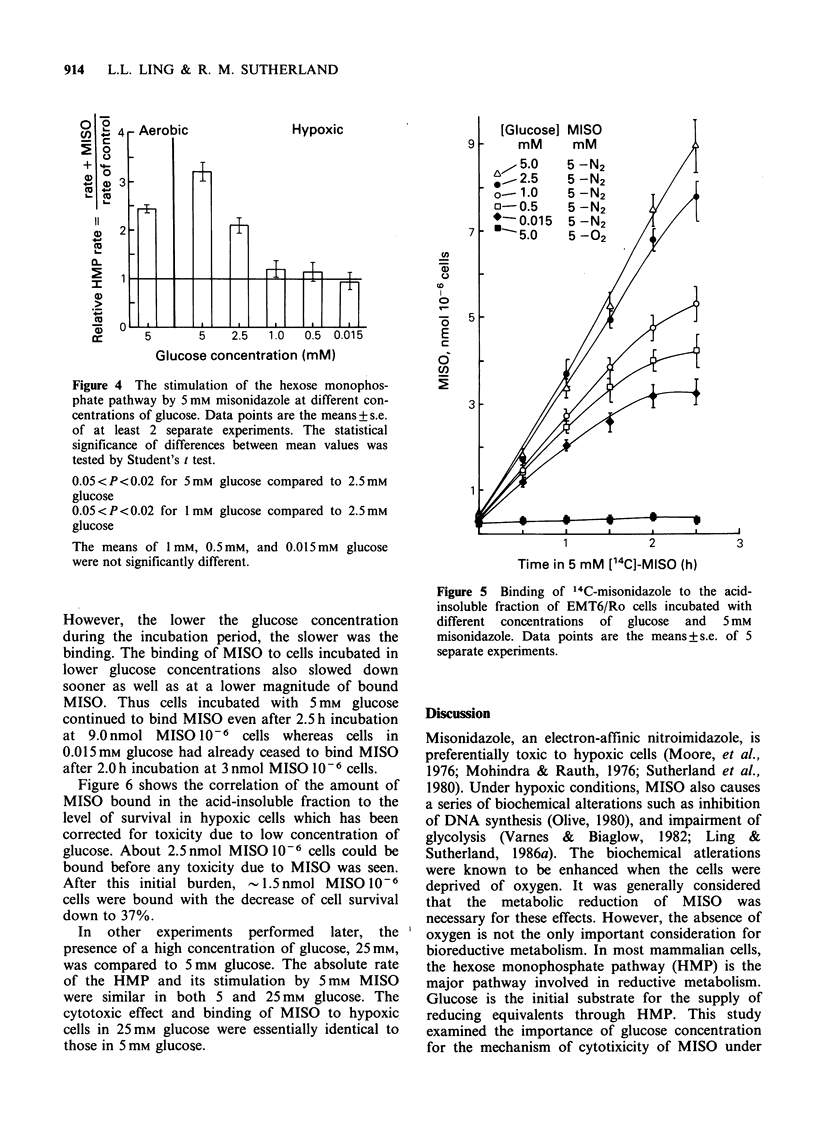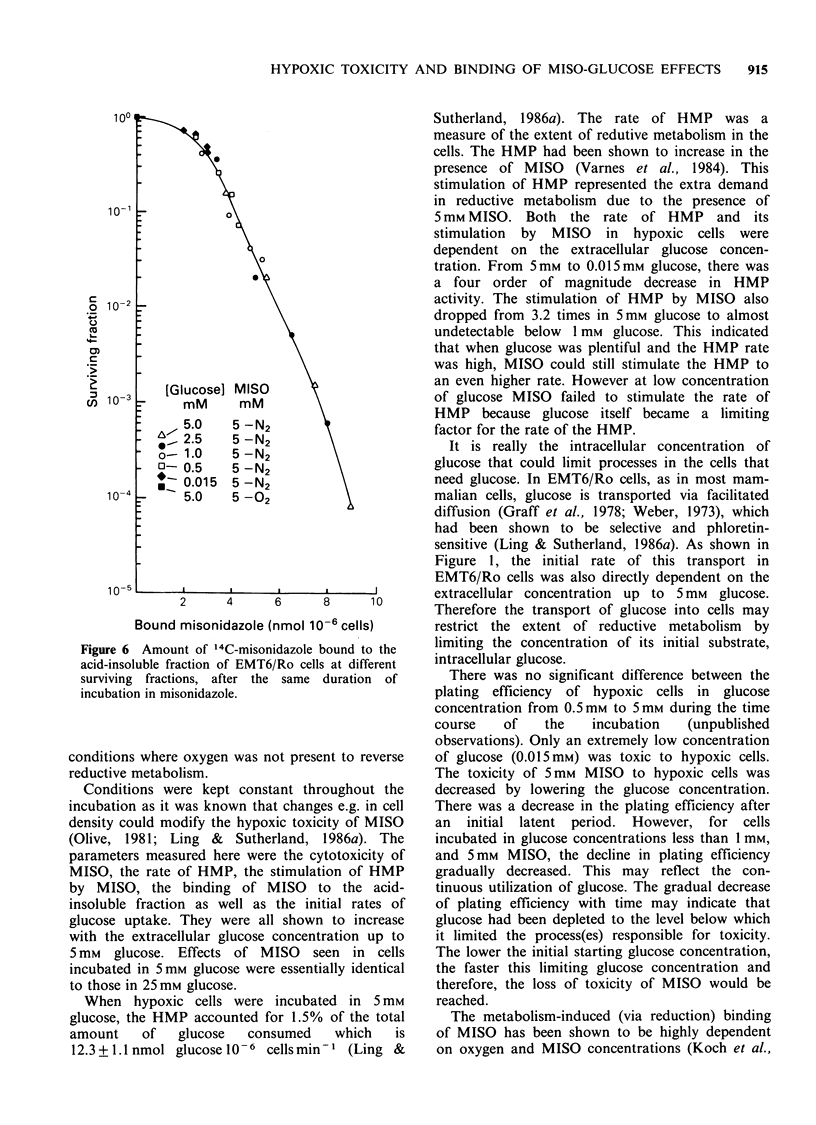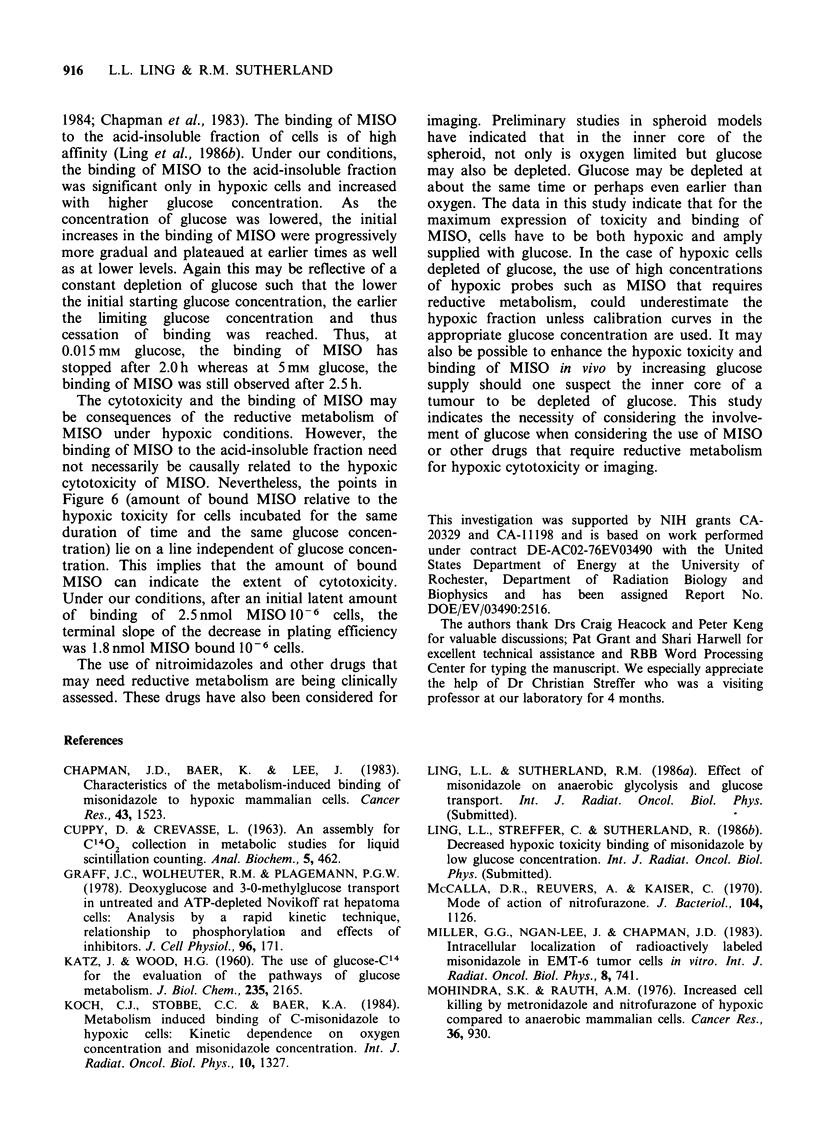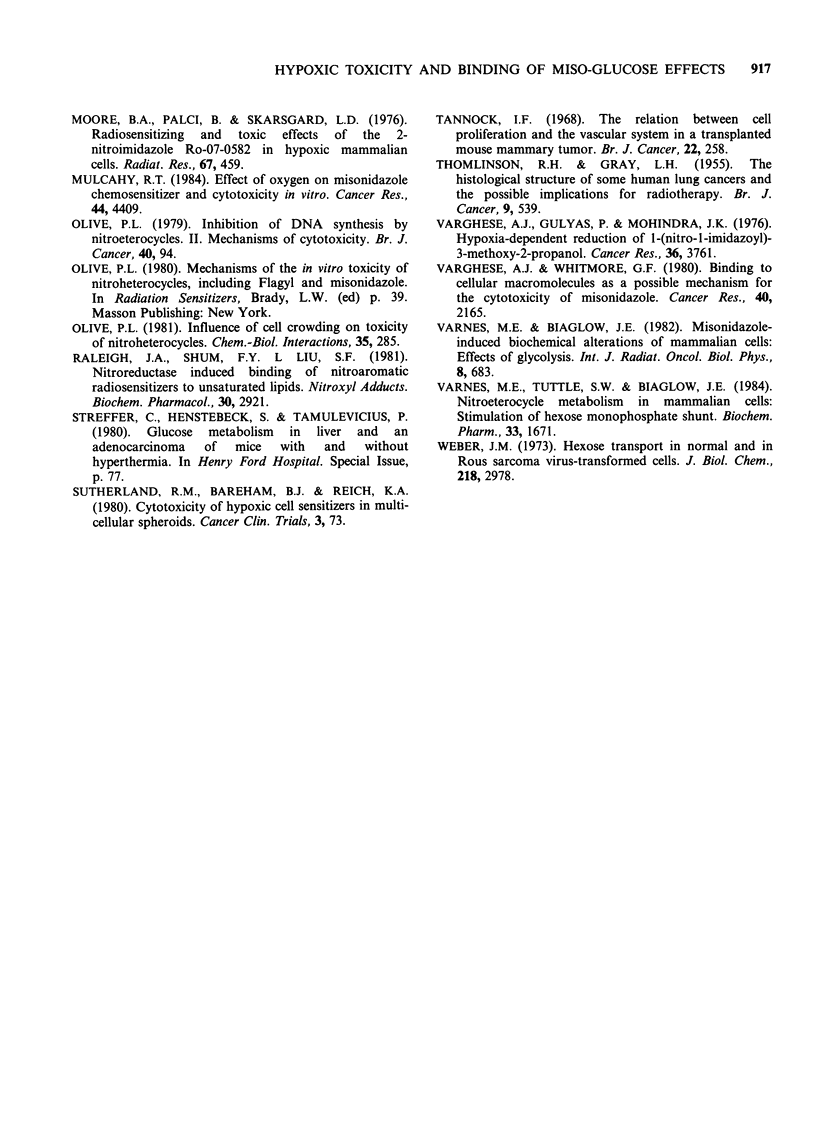# Modulation of the hypoxic toxicity and binding of misonidazole by glucose.

**DOI:** 10.1038/bjc.1986.261

**Published:** 1986-12

**Authors:** L. L. Ling, R. M. Sutherland

## Abstract

The hypoxic toxicity and binding of misonidazole (MISO) requires metabolic reduction. The influence of glucose on the toxicity and binding of MISO was studied because glucose is a major substrate for the supply of NADPH through the hexose monophosphate pathway (HMP). Hypoxic EMT6/Ro cells (10(6) cells ml-1) were incubated with varying concentrations of glucose (0.015 mM to 5 mM). The initial rate of glucose transport was found to increase linearly with the extracellular glucose concentration up to 5 mM (0.038 nmol glucose 10(-6) cells sec-1). About 1.5 percent of the total glucose consumed went through the HMP for hypoxic cells in 5 mM glucose. The rate of HMP progressively decreased as the glucose concentration was lowered. When exposed to 5 mM MISO, the HMP was stimulated. This stimulation declined from 3.2 times in 5 mM glucose to barely detectable below 1 mM glucose. Both the hypoxic toxicity and binding of 5 mM MISO to the acid-insoluble fraction were decreased as the concentration of glucose was lowered. Below 0.5 mM glucose, no significant toxicity due to MISO was observed. There was an initial burden of 2.5 nmol MISO 10(-6) cells bound with little toxicity. After this initial burden, the terminal slope was 1.8 mol MISO bound 10(-6) cells (63 percent decrease in the surviving fraction). These results indicate that glucose concentrations lower than 5 mM can decrease the HMP rate and the toxicity and binding of MISO to hypoxic cells, and imply that calibration curves with normal and low glucose concentrations should be used to estimate the possible hypoxic fraction when MISO is used as a hypoxic probe in vivo.


*Br. J. Cancer* (1986), **54**, 911–917

### Modulation of the hypoxic toxicity and binding of misonidazole by glucose

L.L. Ling & R.M. Sutherland

*Department of Radiation Biology and Biophysics and Cancer Center, Experimental Therapeutics Division, University of Rochester, School of Medicine and Dentistry, Rochester, NY 14642, USA.*

**Summary** The hypoxic toxicity and binding of misonidazole (MISO) requires metabolic reduction. The influence of glucose on the toxicity and binding of MISO was studied because glucose is a major substrate for the supply of NADPH through the hexose menophosphate pathway (HMP). Hypoxic EMT6/Ro cells ($10^6$ cells ml$^{-1}$) were incubated with varying concentrations of glucose (0.015 mM to 5 mM). The initial rate of glucose transport was found to increase linearly with the extracellular glucose concentration up to 5 mM (0.038 nmol glucose $10^{-6}$ cells sec$^{-1}$). About 1.5 percent of the total glucose consumed went through the HMP for hypoxic cells in 5 mM glucose. The rate of HMP progressively decreased as the glucose concentration was lowered. When exposed to 5 mM MISO, the HMP was stimulated. This stimulation declined from 3.2 times in 5 mM glucose to barely detectable below 1 mM glucose. Both the hypoxic toxicity and binding of 5 mM MISO to the acid-insoluble fraction were decreased as the concentration of glucose was lowered. Below 0.5 mM glucose, no significant toxicity due to MISO was observed. There was an initial burden of 2.5 nmol MISO $10^{-6}$ cells bound with little toxicity. After this intial burden, the terminal slope was 1.8 mol MISO bound $10^{-6}$ cells (63 percent decrease in the surviving fraction). These results indicate that glucose concentrations lower than 5 mM can decrease the HMP rate and the toxicity and binding of MISO to hypoxic cells, and imply that calibration curves with normal and low glucose concentrations should be used to estimate the possible hypoxic fraction when MISO is used as a hypoxic probe *in vivo*.

The selective cytoxicity of misonidazole (MISO) towards hypoxic cells has been well established (Moore *et al.*, 1976; Mohindra & Rauth, 1976; Sutherland *et al.*, 1980). Similarly, the binding of MISO with regard to oxygen dependence has been well documented (Miller *et al.*, 1982; Chapman *et al.*, 1983; Koch *et al.*, 1984). Other biochemical atlerations such as inhibitions of glucose consumption, lactate formation (Varnes & Biaglow, 1984; Ling & Sutherland, 1986*a*) and DNA synthesis (Olive, 1979) have also been observed upon incubation of hypoxic mammalian cells with MISO. Metabolic reduction of MISO is considered to be necessary for these biochemical alterations (Chapman *et al.*, 1981; Raleigh *et al.*, 1981; Olive, 1980; McCalla *et al.*, 1970; Varghese *et al.*, 1976). Under hypoxic conditions, MISO is postulated to be metabolically reduced to intermediates capable of binding with a variety of intracellular molecules (Varghese & Whitmore, 1980). The kinetic dependence on oxygen concentration for the toxicity and binding of MISO for several cell lines has been reported (Koch *et al.*, 1984; Mulcahy, 1984).

However, successful metabolic reduction requires not only the necessary enzymes and the absence of oxygen which would reverse reduction, but also substrates to supply reducing equivalents. In most mammalian cells, the hexose monophosphate pathway (HMP) is the major pathway for the supply of reducing equivalents. Glucose is its initial substrate. Concentrations of oxygen and glucose are known to vary among tumours (Thomlinson & Gray, 1955; Tannock, 1968; Streffer *et al.*, 1980). Drugs such as MISO are being investigated for potential therapeutic purposes to overcome the resistance of hypoxic cells to radiation and chemotherapy. Their preferential binding to hypoxic cells may also be useful in locating areas of hypoxia in the body. However for such utilization, it is important that this preferential toxicity and binding to hypoxic cells are independent of factors other than oxygen. It is therefore important to examine the effect of glucose concentration on the toxicity and binding of MISO under hypoxic conditions.

### Materials and methods

### (*a*) *Culture of the cells*

EMT6/Ro cells were maintained as monolayers in continuous exponential growth in BME, (Eagles Basal Medium, Grand Island Biological Co. NY) supplemented with 15% foetal calf serum (Flow Laboratories Inc., MacLean VA), $4.7\times 10^{-2}$ mg ml$^{-1}$ glutamine (Gibco, Grand Island Co., NY), 960 mg ml$^{-1}$ streptomycin, 96 units ml$^{-1}$

Correspondence: R.M. Sutherland.
Received 14 April 1986; and in revised form 5 August 1986.

© The Macmillan Press Ltd., 1986

penicillin. The cells were grown in a humidified incubator at 37°C in an atmosphere of 3% $CO_2$/97% air. The cells were subcultured twice weekly by dissociation with 0.01% lypophilized trypsin (Worthington Biochemical Corp., Freehold, NJ) in sodium citrate buffer, pH 7.2 and routinely checked for mycoplasma contamination.

For these studies, exponential cell cultures were dissociated with 0.01% trypsin for 10 min and concentrated to $10^6$ cells ml$^{-1}$ in BME media with different concentrations of glucose. The cells and MISO were continuously gassed separately with 3% $CO_2$ in nitrogen for 1.5 h at 37°C in glass vials with continuous gentle stirring. After hypoxia of less than 100 ppm oxygen was induced (Mulcahy, 1984), hypoxic MISO was added to the cell suspension. At different times, the following measurements were made.

*(b) Survival*

Survival was determined by the colony forming assay. The cells were seeded into triplicate plastic cultures dishes at concentrations to give 50 colonies. Normally, triplicates of two dilutions were set for each experimental point determined. After 11 days the plates were stained with methylene blue and colonies of greater than 50 cells were scored.

*(c) Hexose monophosphate pathway*

Hexose monophosphate pathway (HMP) activity was determined by the release of $^{14}CO_2$ from C-1 glucose (Cuppy & Crevasse, 1963; Katz & Wood, 1960). One $\mu$Ci of the labelled glucose was added for every 10 ml of media. After hypoxia of less than 100 ppm oxygen was achieved, hypoxic cells were added to the media and the vials tightly sealed. At different times, $^{14}CO_2$ was released from the cell suspension by acidification with 0.2 ml 6N HCl. Air was flushed through the vial into 1 M KOH. Constant flushing with serial tubes of 1 M KOH for different times, and the capacity to quantitatively account for all the radioactivity added at the end of the experiment determine that complete trapping of $^{14}CO_2$ is accomplished within 0.5 h of flushing. After flushing for 0.5 h, an aliquot (1 ml) of the KOH was counted in 10 ml of scintillation fluid (Scintiverse, Fischer Company, USA), to determine the amount of $^{14}CO_2$ trapped in it.

*(d) Binding of $^{14}$C-MISO*

Binding of $^{14}$C-MISO (labelled at C-2 of the imidazole ring) to cells was determined by adding $^{14}$C-MISO to a final concentration of 5 mM (specific activity 0.2 mCi mmol$^{-1}$). After different times of incubation, 1 ml of the cell suspension was removed and spun down. The pellet was washed with 1 ml of ice cold saline solution before resuspension in 1 ml of ice-cold 10% trichloroacetic acid (TCA). After 10 min, the TCA precipitate was washed once with 1 ml of ice cold 10% TCA and then counted in 5 ml of scintillation fluid (Scintiverse, Fisher Company, USA).

*(e) Glucose transport*

Glucose transport was measured with the D-glucose analog, 3-0-methyl-D-glucose (3-OMG), which is transported into the cells in the same way as D-glucose (Graff *et al.*, 1978; Weber, 1973). The cells were incubated in normal growth media and [$^{14}$C]-3-0-methyl-D-glucose (0.3 $\mu$Ci ml$^{-1}$, Amersham; 120 $\mu$Ci mmol$^{-1}$) for various lengths of time and rapidly separated from their radioactive media by spinning through a mixture of n-butyl-phthalate:Mazola corn oil (4:1 mixture). The cell pellets were then dried and counted. Incubations were carried out at 15 sec intervals up to a minute to obtain an initial rate of uptake.

### Results

The intracellular concentration of glucose could limit processes that need glucose in the cells. In $EMT_6$/Ro cells, glucose had been shown to be transported via a facilitated diffusion that is both selective and phloretin-sensitive (Ling & Sutherland, 1986*a*). When the glucose analog, 3-0-methyl-D-glucose, is used in such transport experiments, an equilibrium of intracellular concentration equivalent to the extracellular concentration is achieved within a minute. Figure 1 shows that this transport of glucose is also dependent on the glucose concentrations in EMT6/Ro cells. Similar results are observed for both aerobic and acutely hypoxic EMT6/Ro cells. The initial rates of glucose uptake depend on the extracellular concentration of glucose and increased linearly with glucose concentration up to 5 mM glucose. Increasing the concentration 5-fold to 25 mM glucose did not further increase the rate of uptake. Thus the rates of activity of glucose metabolic pathways which could affect the redox balance of the cells and therefore, reductive metabolism of MISO, would be expected to vary significantly over the range of 0 to 5 mM glucose.

Figure 2 shows the effect of lowering glucose over this concentration range on the hypoxic toxicity of 5 mM MISO. As the concentration of glucose was lowered, hypoxic toxicity due to 5 mM MISO progressively lessened. This was seen primarily as a decrease in the terminal slope of killing after an initial latent period. The absence of glucose was itself slightly toxic, decreasing the

**Figure 1** The initial rates of glucose uptake at different concentrations of glucose. Data points are the means ± s.e. of 3 separate experiments.

**Figure 2** Surviving fraction of hypoxic EMT6/Ro cells ($10^6$ cells $ml^{-1}$) incubated with different concentrations of glucose and misonidazole. The data were corrected for the plating efficiency of the cells at the start of the experiment. Data points are the means ± s.e. of 3 separate experiments.

surviving fraction to 10% after 2.5 h of hypoxic incubation. Below 0.5 mM glucose, no significant toxicity due to MISO was observed.

For hypoxic EMT6/Ro cells in 5 mM glucose, ~1.5% of the total glucose was consumed through the hexose monophosphate pathway (HMP). Figure 3 shows the rates of HMP in EMT6/Ro cells. For cells in 5 mM glucose, the rate of HMP was slightly decreased by the absence of oxygen and stimulated by the presence of 5 mM MISO. The absolute rate of HMP was dependent on the concentration of glucose supplied. It progressively decreased as the concentration of glucose decreased.

The presence of 5 mM MISO was found to stimulate the HMP rate in both aerobic and hypoxic conditions. This stimulation was also dependent on glucose concentration (Figure 4). Under hypoxic condition, this stimulation decreased from 3.2 times in 5 mM glucose to barely detectable in 1 mM glucose.

Figure 5 shows that the binding of $^{14}$C-MISO to the acid-insoluble fraction in hypoxic cells was similarly dependent on glucose concentration. Binding was significant only in hypoxic cells. There was an initial increase in binding before a gradual cessation for most concentrations of glucose used.

**Figure 3** The rates of hexose monophosphate pathway of EMT6/Ro cells incubated with different concentrations of glucose and 5 mM misonidazole. Data points are the means ± s.e. of at least 2 separate experiments.

**Figure 4** The stimulation of the hexose monophosphate pathway by 5 mM misonidazole at different concentrations of glucose. Data points are the means ± s.e. of at least 2 separate experiments. The statistical significance of differences between mean values was tested by Student's *t* test.

$0.05 < P < 0.02$ for 5 mM glucose compared to 2.5 mM glucose

$0.05 < P < 0.02$ for 1 mM glucose compared to 2.5 mM glucose

The means of 1 mM, 0.5 mM, and 0.015 mM glucose were not significantly different.

**Figure 5** Binding of $^{14}$C-misonidazole to the acid-insoluble fraction of EMT6/Ro cells incubated with different concentrations of glucose and 5 mM misonidazole. Data points are the means ± s.e. of 5 separate experiments.

However, the lower the glucose concentration during the incubation period, the slower was the binding. The binding of MISO to cells incubated in lower glucose concentrations also slowed down sooner as well as at a lower magnitude of bound MISO. Thus cells incubated with 5 mM glucose continued to bind MISO even after 2.5 h incubation at 9.0 nmol MISO $10^{-6}$ cells whereas cells in 0.015 mM glucose had already ceased to bind MISO after 2.0 h incubation at 3 nmol MISO $10^{-6}$ cells.

Figure 6 shows the correlation of the amount of MISO bound in the acid-insoluble fraction to the level of survival in hypoxic cells which has been corrected for toxicity due to low concentration of glucose. About 2.5 nmol MISO $10^{-6}$ cells could be bound before any toxicity due to MISO was seen. After this initial burden, ~1.5 nmol MISO $10^{-6}$ cells were bound with the decrease of cell survival down to 37%.

In other experiments performed later, the presence of a high concentration of glucose, 25 mM, was compared to 5 mM glucose. The absolute rate of the HMP and its stimulation by 5 mM MISO were similar in both 5 and 25 mM glucose. The cytotoxic effect and binding of MISO to hypoxic cells in 25 mM glucose were essentially identical to those in 5 mM glucose.

### Discussion

Misonidazole, an electron-affinic nitroimidazole, is preferentially toxic to hypoxic cells (Moore, *et al.*, 1976; Mohindra & Rauth, 1976; Sutherland *et al.*, 1980). Under hypoxic conditions, MISO also causes a series of biochemical alterations such as inhibition of DNA synthesis (Olive, 1980), and impairment of glycolysis (Varnes & Biaglow, 1982; Ling & Sutherland, 1986*a*). The biochemical atlerations were known to be enhanced when the cells were deprived of oxygen. It was generally considered that the metabolic reduction of MISO was necessary for these effects. However, the absence of oxygen is not the only important consideration for bioreductive metabolism. In most mammalian cells, the hexose monophosphate pathway (HMP) is the major pathway involved in reductive metabolism. Glucose is the initial substrate for the supply of reducing equivalents through HMP. This study examined the importance of glucose concentration for the mechanism of cytotixicity of MISO under

**Figure 6** Amount of $^{14}$C-misonidazole bound to the acid-insoluble fraction of EMT6/Ro cells at different surviving fractions, after the same duration of incubation in misonidazole.

conditions where oxygen was not present to reverse reductive metabolism.

Conditions were kept constant throughout the incubation as it was known that changes e.g. in cell density could modify the hypoxic toxicity of MISO (Olive, 1981; Ling & Sutherland, 1986*a*). The parameters measured here were the cytotoxicity of MISO, the rate of HMP, the stimulation of HMP by MISO, the binding of MISO to the acid-insoluble fraction as well as the initial rates of glucose uptake. They were all shown to increase with the extracellular glucose concentration up to 5 mM glucose. Effects of MISO seen in cells incubated in 5 mM glucose were essentially identical to those in 25 mM glucose.

When hypoxic cells were incubated in 5 mM glucose, the HMP accounted for 1.5% of the total amount of glucose consumed which is $12.3 \pm 1.1$ nmol glucose $10^{-6}$ cells min$^{-1}$ (Ling & Sutherland, 1986*a*). The rate of HMP was a measure of the extent of redutive metabolism in the cells. The HMP had been shown to increase in the presence of MISO (Varnes *et al.*, 1984). This stimulation of HMP represented the extra demand in reductive metabolism due to the presence of 5 mM MISO. Both the rate of HMP and its stimulation by MISO in hypoxic cells were dependent on the extracellular glucose concentration. From 5 mM to 0.015 mM glucose, there was a four order of magnitude decrease in HMP activity. The stimulation of HMP by MISO also dropped from 3.2 times in 5 mM glucose to almost undetectable below 1 mM glucose. This indicated that when glucose was plentiful and the HMP rate was high, MISO could still stimulate the HMP to an even higher rate. However at low concentration of glucose MISO failed to stimulate the rate of HMP because glucose itself became a limiting factor for the rate of the HMP.

It is really the intracellular concentration of glucose that could limit processes in the cells that need glucose. In EMT6/Ro cells, as in most mammalian cells, glucose is transported via facilitated diffusion (Graff *et al.*, 1978; Weber, 1973), which had been shown to be selective and phloretin-sensitive (Ling & Sutherland, 1986*a*). As shown in Figure 1, the initial rate of this transport in EMT6/Ro cells was also directly dependent on the extracellular concentration up to 5 mM glucose. Therefore the transport of glucose into cells may restrict the extent of reductive metabolism by limiting the concentration of its initial substrate, intracellular glucose.

There was no significant difference between the plating efficiency of hypoxic cells in glucose concentration from 0.5 mM to 5 mM during the time course of the incubation (unpublished observations). Only an extremely low concentration of glucose (0.015 mM) was toxic to hypoxic cells. The toxicity of 5 mM MISO to hypoxic cells was decreased by lowering the glucose concentration. There was a decrease in the plating efficiency after an initial latent period. However, for cells incubated in glucose concentrations less than 1 mM, and 5 mM MISO, the decline in plating efficiency gradually decreased. This may reflect the continuous utilization of glucose. The gradual decrease of plating efficiency with time may indicate that glucose had been depleted to the level below which it limited the process(es) responsible for toxicity. The lower the initial starting glucose concentration, the faster this limiting glucose concentration and therefore, the loss of toxicity of MISO would be reached.

The metabolism-induced (via reduction) binding of MISO has been shown to be highly dependent on oxygen and MISO concentrations (Koch *et al.*,

1984; Chapman *et al.*, 1983). The binding of MISO to the acid-insoluble fraction of cells is of high affinity (Ling *et al.*, 1986*b*). Under our conditions, the binding of MISO to the acid-insoluble fraction was significant only in hypoxic cells and increased with higher glucose concentration. As the concentration of glucose was lowered, the initial increases in the binding of MISO were progressively more gradual and plateaued at earlier times as well as at lower levels. Again this may be reflective of a constant depletion of glucose such that the lower the initial starting glucose concentration, the earlier the limiting glucose concentration and thus cessation of binding was reached. Thus, at 0.015 mM glucose, the binding of MISO has stopped after 2.0 h whereas at 5 mM glucose, the binding of MISO was still observed after 2.5 h.

The cytotoxicity and the binding of MISO may be consequences of the reductive metabolism of MISO under hypoxic conditions. However, the binding of MISO to the acid-insoluble fraction need not necessarily be causally related to the hypoxic cytotoxicity of MISO. Nevertheless, the points in Figure 6 (amount of bound MISO relative to the hypoxic toxicity for cells incubated for the same duration of time and the same glucose concentration) lie on a line independent of glucose concentration. This implies that the amount of bound MISO can indicate the extent of cytotoxicity. Under our conditions, after an initial latent amount of binding of 2.5 nmol MISO $10^{-6}$ cells, the terminal slope of the decrease in plating efficiency was 1.8 nmol MISO bound $10^{-6}$ cells.

The use of nitroimidazoles and other drugs that may need reductive metabolism are being clinically assessed. These drugs have also been considered for imaging. Preliminary studies in spheroid models have indicated that in the inner core of the spheroid, not only is oxygen limited but glucose may also be depleted. Glucose may be depleted at about the same time or perhaps even earlier than oxygen. The data in this study indicate that for the maximum expression of toxicity and binding of MISO, cells have to be both hypoxic and amply supplied with glucose. In the case of hypoxic cells depleted of glucose, the use of high concentrations of hypoxic probes such as MISO that requires reductive metabolism, could underestimate the hypoxic fraction unless calibration curves in the appropriate glucose concentration are used. It may also be possible to enhance the hypoxic toxicity and binding of MISO *in vivo* by increasing glucose supply should one suspect the inner core of a tumour to be depleted of glucose. This study indicates the necessity of considering the involvement of glucose when considering the use of MISO or other drugs that require reductive metabolism for hypoxic cytotoxicity or imaging.

This investigation was supported by NIH grants CA-20329 and CA-11198 and is based on work performed under contract DE-AC02-76EV03490 with the United States Department of Energy at the University of Rochester, Department of Radiation Biology and Biophysics and has been assigned Report No. DOE/EV/03490:2516.

The authors thank Drs Craig Heacock and Peter Keng for valuable discussions; Pat Grant and Shari Harwell for excellent technical assistance and RBB Word Processing Center for typing the manuscript. We especially appreciate the help of Dr Christian Streffer who was a visiting professor at our laboratory for 4 months.

### References

CHAPMAN, J.D., BAER, K. & LEE, J. (1983). Characteristics of the metabolism-induced binding of misonidazole to hypoxic mammalian cells. *Cancer Res.*, **43**, 1523.

CUPPY, D. & CREVASSE, L. (1963). An assembly for $C^{14}O_2$ collection in metabolic studies for liquid scintillation counting. *Anal. Biochem.*, **5**, 462.

GRAFF, J.C., WOLHEUTER, R.M. & PLAGEMANN, P.G.W. (1978). Deoxyglucose and 3-0-methylglucose transport in untreated and ATP-depleted Novikoff rat hepatoma cells: Analysis by a rapid kinetic technique, relationship to phosphorylation and effects of inhibitors. *J. Cell Physiol.*, **96**, 171.

KATZ, J. & WOOD, H.G. (1960). The use of glucose-$C^{14}$ for the evaluation of the pathways of glucose metabolism. *J. Biol. Chem.*, **235**, 2165.

KOCH, C.J., STOBBE, C.C. & BAER, K.A. (1984). Metabolism induced binding of C-misonidazole to hypoxic cells: Kinetic dependence on oxygen concentration and misonidazole concentration. *Int. J. Radiat. Oncol. Biol. Phys.*, **10**, 1327.

LING, L.L. & SUTHERLAND, R.M. (1986*a*). Effect of misonidazole on anaerobic glycolysis and glucose transport. *Int. J. Radiat. Oncol. Biol. Phys.* (Submitted).

LING, L.L., STREFFER, C. & SUTHERLAND, R. (1986*b*). Decreased hypoxic toxicity binding of misonidazole by low glucose concentration. *Int. J. Radiat. Oncol. Biol. Phys.* (Submitted).

McCALLA, D.R., REUVERS, A. & KAISER, C. (1970). Mode of action of nitrofurazone. *J. Bacteriol.*, **104**, 1126.

MILLER, G.G., NGAN-LEE, J. & CHAPMAN, J.D. (1983). Intracellular localization of radioactively labeled misonidazole in EMT-6 tumor cells *in vitro*. *Int. J. Radiat. Oncol. Biol. Phys.*, **8**, 741.

MOHINDRA, S.K. & RAUTH, A.M. (1976). Increased cell killing by metronidazole and nitrofurazone of hypoxic compared to anaerobic mammalian cells. *Cancer Res.*, **36**, 930.

MOORE, B.A., PALCI, B. & SKARSGARD, L.D. (1976). Radiosensitizing and toxic effects of the 2-nitroimidazole Ro-07-0582 in hypoxic mammalian cells. *Radiat. Res.*, **67**, 459.

MULCAHY, R.T. (1984). Effect of oxygen on misonidazole chemosensitizer and cytotoxicity *in vitro*. *Cancer Res.*, **44**, 4409.

OLIVE, P.L. (1979). Inhibition of DNA synthesis by nitroeterocycles. II. Mechanisms of cytotoxicity. *Br. J. Cancer*, **40**, 94.

OLIVE, P.L. (1980). Mechanisms of the *in vitro* toxicity of nitroheterocycles, including Flagyl and misonidazole. In *Radiation Sensitizers*, Brady, L.W. (ed) p. 39. Masson Publishing: New York.

OLIVE, P.L. (1981). Influence of cell crowding on toxicity of nitroheterocycles. *Chem.-Biol. Interactions*, **35**, 285.

RALEIGH, J.A., SHUM, F.Y. L LIU, S.F. (1981). Nitroreductase induced binding of nitroaromatic radiosensitizers to unsaturated lipids. *Nitroxyl Adducts. Biochem. Pharmacol.*, **30**, 2921.

STREFFER, C., HENSTEBECK, S. & TAMULEVICIUS, P. (1980). Glucose metabolism in liver and an adenocarcinoma of mice with and without hyperthermia. In *Henry Ford Hospital*. Special Issue, p. 77.

SUTHERLAND, R.M., BAREHAM, B.J. & REICH, K.A. (1980). Cytotoxicity of hypoxic cell sensitizers in multicellular spheroids. *Cancer Clin. Trials*, **3**, 73.

TANNOCK, I.F. (1968). The relation between cell proliferation and the vascular system in a transplanted mouse mammary tumor. *Br. J. Cancer*, **22**, 258.

THOMLINSON, R.H. & GRAY, L.H. (1955). The histological structure of some human lung cancers and the possible implications for radiotherapy. *Br. J. Cancer*, **9**, 539.

VARGHESE, A.J., GULYAS, P. & MOHINDRA, J.K. (1976). Hypoxia-dependent reduction of 1-(nitro-1-imidazoyl)-3-methoxy-2-propanol. *Cancer Res.*, **36**, 3761.

VARGHESE, A.J. & WHITMORE, G.F. (1980). Binding to cellular macromolecules as a possible mechanism for the cytotoxicity of misonidazole. *Cancer Res.*, **40**, 2165.

VARNES, M.E. & BIAGLOW, J.E. (1982). Misonidazole-induced biochemical alterations of mammalian cells: Effects of glycolysis. *Int. J. Radiat. Oncol. Biol. Phys.*, **8**, 683.

VARNES, M.E., TUTTLE, S.W. & BIAGLOW, J.E. (1984). Nitroeterocycle metabolism in mammalian cells: Stimulation of hexose monophosphate shunt. *Biochem. Pharm.*, **33**, 1671.

WEBER, J.M. (1973). Hexose transport in normal and in Rous sarcoma virus-transformed cells. *J. Biol. Chem.*, **218**, 2978.